# Preparing for a COVID-19 pandemic: a review of operating room outbreak response measures in a large tertiary hospital in India

**DOI:** 10.1186/s42077-021-00150-w

**Published:** 2021-04-12

**Authors:** Nazia Nazir, Savita Gupta

**Affiliations:** Department of Anaesthesiology and Critical Care, Government Institute of Medical Sciences, 15/6; Block F; Gautam Buddha University Campus, Greater Noida, Uttar Pradesh India

## Introduction

WHO declared the novel coronavirus disease 2019 (COVID-19) as pandemic on 11 March 2020 because of its rapid worldwide spread (World Health Organization, 2020a). India’s first coronavirus case was detected on 10 January 2020 in Kerala (Narasimhan, 2020). Since then, the disease is showing an increasing trend. As of 23 September 2020, India has recorded 5.65 million positive cases with 90.020 mortalities (Home, n.d.). Because the virus is novel to human beings, and there is no vaccine yet available, every individual is susceptible and can become infected. Healthcare workers are at high risk, and unfortunately, more than 3000 healthcare workers in China have been infected. Anesthesiologists are among healthcare workers who are at an even higher risk of becoming infected because of their close contact with infected patients and high potential of exposure to respiratory droplets or aerosol from their patients’ airways.

Our hospital was declared as a Corona Care Centre and started screening and cough OPD and sampling since 27 January 2020 while the first case positive case was admitted in our institute on 4 March 2020. There have been several reports of anesthesiologists being infected after providing tracheal intubation for confirmed COVID-19 patients, although the exact number of is not known. Our department of anesthesia started preparing for the COVID-19 pandemic on two fronts. Preparation of a COVID operating room (OR) for a suspected or confirmed case of COVID undergoing emergency surgery and preparation of COVID ICU for suspected and confirmed COVID cases.

The operating room, being a busy environment, further increases the risk of contracting the infection. Therefore, urgent development of safe medical practices and infection prevention protocols for the perioperative management of patients with COVID-19 was required.

The following are the preparations and standard operating procedures (SOP) adopted at our institute.

### Hospital measures related to anesthesia and surgery

#### Increasing capacity

To cope with the anticipated increased influx of COVID-19 patients, we decided to reduce elective surgery to avoid non-urgent hospitalization and minimize elective surgeries (Press Information Bureau Government of India Ministry of Health and Family Welfare, 2020). Day surgeries and other emergency operations continued as normal. At our end, we also increased the turnaround time between the elective surgeries to accommodate additional infection prevention practices.

### Management of patients, visitors, and staff

Thermal scanners and hand sanitizers were installed at the entry of hospital. All patients coming to the hospital were screened using a standard questionnaire and those who fulfilled the criteria for suspected severe acute respiratory syndrome coronavirus 2 (SARS-CoV-2) as per Clinical Management of COVID-19 triage were isolated, referred to an infectious diseases specialist, and tested for the virus (World Health Organization, 2020b). Patient attendants and any visitor were also restricted. We also separated staff caring for COVID-19 patients and those caring for other patients to reduce the risk of in-hospital (Chee et al., 2004). Any elective surgery was postponed if the patient had travelled to affected areas. If patient not under GA then patient should wear a surgical mask.

### Preparation

The patients were requested to remove any facial hair to ensure an adequate seal with a facemask.

### Operating theatre setup (https://www.asahq.org/about-asa/governance-and-committees/asa-committees/committee-on-occupational-health/coronavirus, 2020; https://www.apsf.org/news-updates/perioperative-considerations-for-the-2019-novel-coronavirus-covid-19/, 2020; Chen et al., 2020)

We removed any unnecessary equipment from the theatre to prevent cross-contamination. A runner was posted outside the room to pass equipment into the theatre. Any contaminated waste was placed in a large yellow bin for and separate bin (not yellow) to place soiled reusable equipment temporarily until cleaned.

Operating theatre departments identified operating theatre(s) to be used to manage COVID-19 patients ensuring a clear protocol for patient flow into the operating theatre. Neutral pressure room was used for performing aerosolizing procedures.

**Fig. 1:**
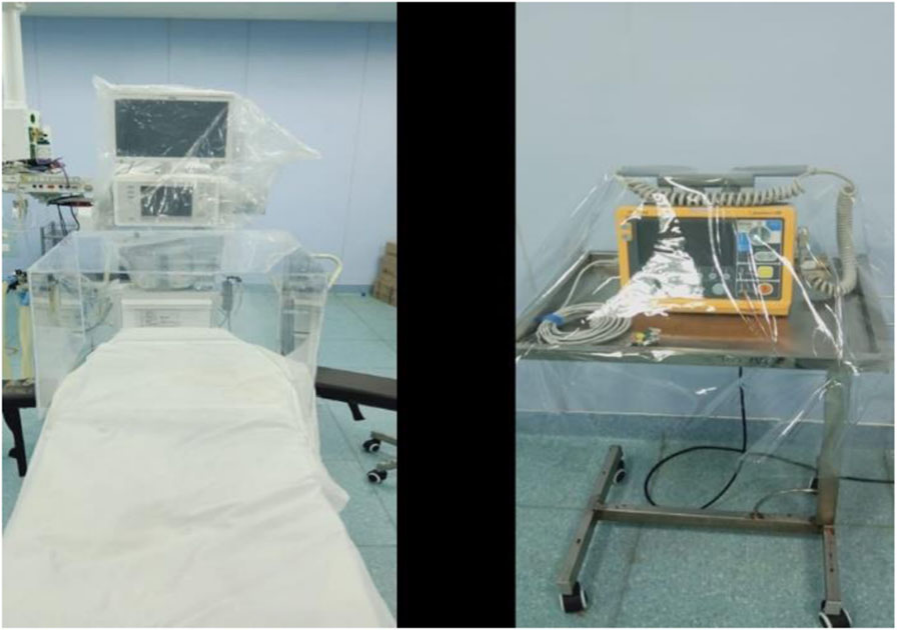
Covering of equipment with plastic sheets

At our institute, we were fortunate to have a modular OR which was not connected to an Air Handling Unit (AHU). This OR was not being used previously. Being an ‘Airborne Isolation Room, this OR was converted into a COVID OR. An anesthesia workstation and all the necessary equipment were shifted. All the equipment was covered with transparent plastic sheets to prevent unnecessary contamination (Fig. 1). The disposable breathing circuit was attached with bacterial/viral filters at both the y piece and expiratory limb to prevent contamination of machine. SOP to dispose off disposables like circuit, soda lime, HME, and viral filters were made.

On arrival at the operation theatre, we ensured a surgical mask to be placed on the patient if not already done so and escorted them directly to theatre to minimize exposure to staff and other patients en route.

A plan was made in place to ensure that critical cases requiring immediate care such as caesarean section, severe hemorrhage or airway emergencies were cared for, including consideration of location of surgery (designated OR with signs posted on the doors to minimize staff entry), immediate availability of PPE, and team formulation.

**Fig. 2:**
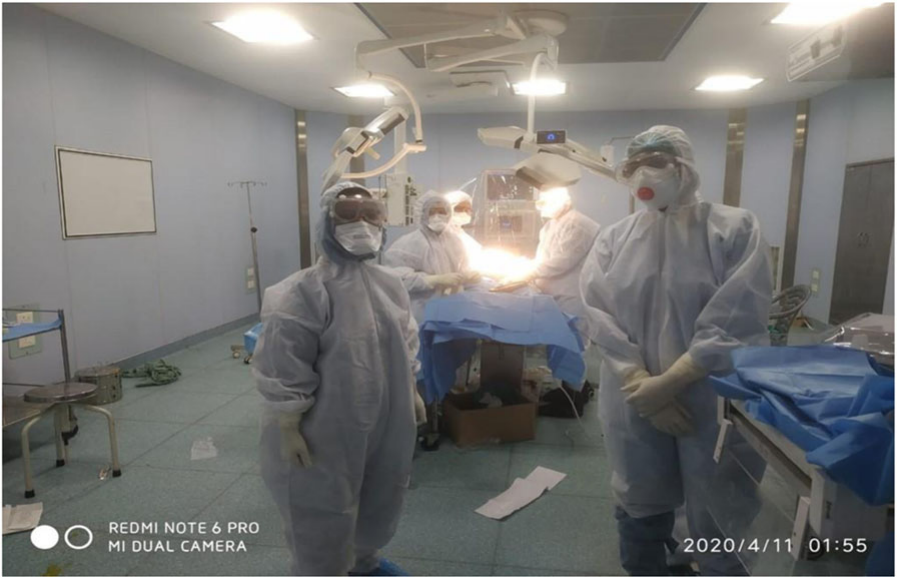
Anesthesia and surgical team in COVID OT

We formulated anesthetic teams who were preferentially be called to lead the airway management of patients with COVID-19. This had some advantages in reducing the number of staff potentially exposed, avoiding staff who may be ‘high-risk’ (such as those with comorbidities), and fostering expertise among smaller groups (Fig. 2).

### Setting up donning and doffing rooms

The donning and doffing rooms were marked in vicinity of OR to minimize contamination and spread. The rooms were again airborne isolation rooms. Posters were kept in both rooms to point the steps of both. Simulation testing of the processes for critical and non-critical cases, including airway management were undertaken to identify system issues and gaps. The importance of doffing with minimal generation of aerosol was stressed upon.

### Manpower

The protocol of having minimum manpower inside the OR with minimum movement was made. All healthcare personnel were continuously trained for dealing with any foreseeable emergencies. Repeated training of anesthesiologists and technicians with the use of patient screen over mannikins was carried out to familiarize the process.

### Airway management (Wax & Christian, 2020; Zucco et al., 2020; World Health Organization, n.d.)

Since the anesthesiologists are at the highest risk of becoming infected while dealing with patients’ airways additional protective measures were planned to minimize the exposure. Airway management was performed by the most senior anesthesiologist available in the team, with full-PPE precautions.

The airway management was aimed at minimizing disconnection, aerosolization, and exposure of staff to viral transmission. Intubation was recommended rather than use of supraglottic airways unless required for airway ‘rescue.’

**Fig. 3:**
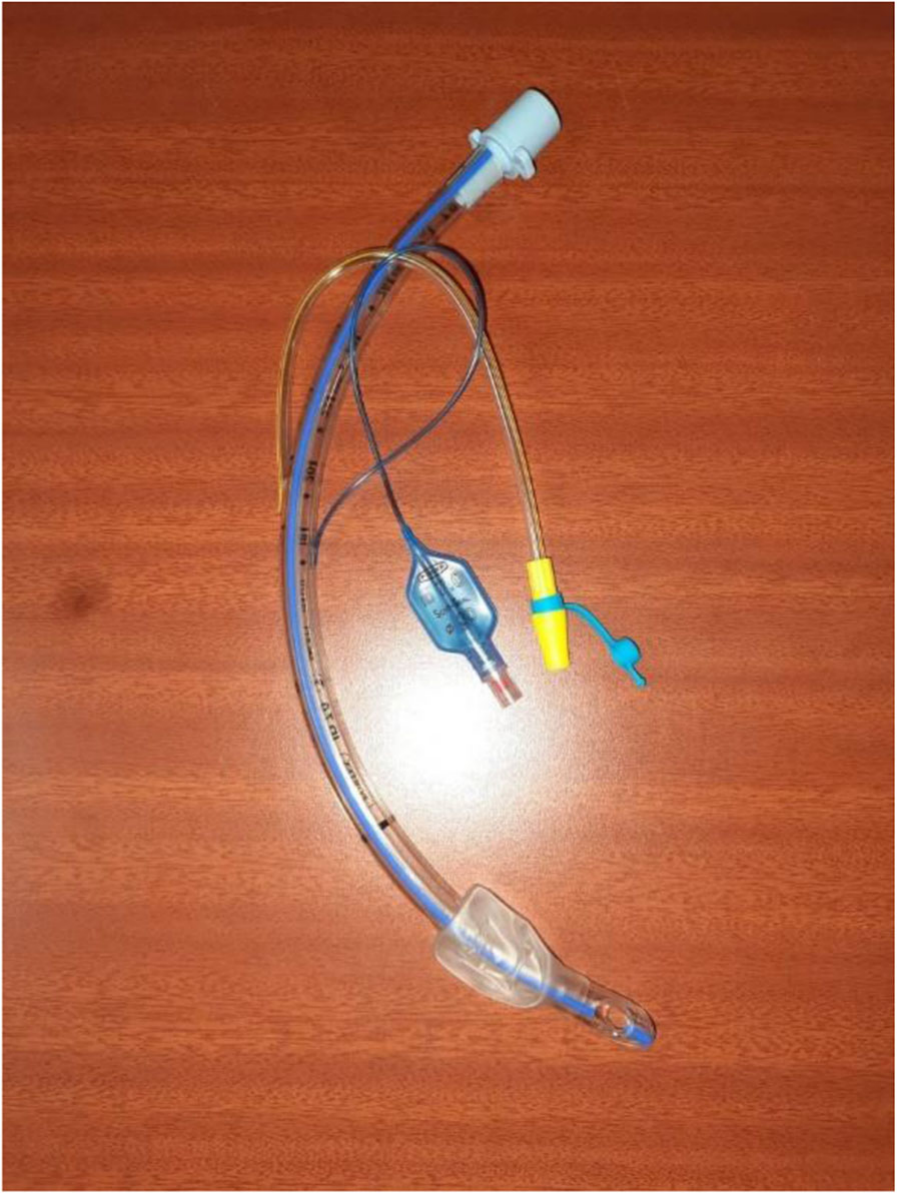
Endotracheal tube with side suction port

- Ensured adequate time for reviewing the intubation plan and for donning PPE.
- A transparent patient screen (custom made according to our need) was prepared using acrylic sheets (Fig. 3).
- For patients requiring supplemental oxygen, disposable face masks with rebreathing bag are to be used instead of nasal prongs.
- Preloading of the ETT onto a stylet or bougie was preferred to improve the chance of successful intubation first time, though there may be a risk of increasing droplet spread on removal—use of ‘gauze-wipe’ technique was done.
- Use of a ‘rapid sequence induction’ procedure was recommended to avoid manual ventilation if possible.
- We used muscle relaxant and allowed for full effect before intubation.
- A video laryngoscope with disposable blades to be used for intubation (Birnbach et al., 2015).
- Endotracheal tubes with side port for suctioning to be placed.
- Closed suction system to be used.
- Any procedure causing increase in aerosolization like high flow nasal cannula, awake fiber optic intubation, and bag masking are to be avoided.
- Once the ET tube was inserted, cuff was sufficiently inflated before reconnecting the filter to the ET tube and connecting to the breathing circuit and test ventilating.
- Re-sheath of the laryngoscope blade immediately post-intubation was done and all used equipment was sealed in a double zip-locked bag.
- If the use of PPE has included ‘double gloving’, the outside gloves were removed as soon as successful intubation was confirmed.

### Extubation

Like intubation, extubation was also aimed to avoid aerosolization and minimize staff exposure. Techniques considered included spontaneously breathing deep extubation to reduce coughing and LMA exchange using a close circuit to avoid coughing/straining (Munoz-Price et al., 2019).

### Transfers

For patients requiring transfer to ICU.

- We minimized the number and duration of breathing circuit disconnections.
- When disconnecting and reconnecting to a ventilator, we left the filter attached to patient end. The endotracheal tube was clamped and the ventilator disabled to prevent aerosolization.
- A transport ventilator or self-inflating bag with a filter was used.
- Use of inline (closed) suction was done.

### Post procedure

- Discarded filters and breathing circuits were disposed off in yellow bags.
- Operating theatre was cleaned and sanitized as per hospital policy after each procedure.

### Other important interventions

#### Administrative

We also made a policy of treating free of cost of any healthcare worker who pick up infection while treating patients. Non-essential audits of our hospital by various regulators and accreditation agencies like NABH was postponed. We also ensured social distancing in our premises by putting up bilingual pamphlets at various strategic locations. Leave of all kinds (except under emergency and unavoidable circumstances) were canceled immediately.

### IEC activities

Patients were educated about cough etiquette, do’s and don’ts, proper use of masks instead of using them indiscriminately and inefficiently; and personal hygiene by our infection control nurses. At various locations, posters were put to increase awareness among patients on do’s and don’ts regarding COVID 19.

## Data Availability

Not applicable

## Abbreviations

OPD: Out-patient department
COVID: Coronavirus disease
OR: Operating room
ICU: Intensive care unit
SOP: Standard operating procedure
SARS-CoV-2: Severe acute respiratory syndrome coronavirus 2
GA: General anesthesia
AHU: Air handling unit
HME: Heat moisture exchanger
PPE: Personal protective equipment
ET Tube: Endotracheal tube
LMA: Laryngeal mask airway
NABH: National accreditation board of hospitals
IEC: Institutional ethics committee
